# CCR2^+^ migratory macrophages with M1 status are the early-responders in the cornea of HSV-1 infected mice

**DOI:** 10.1371/journal.pone.0215727

**Published:** 2019-04-18

**Authors:** Dhong Hyun Lee, Ujjaldeep Jaggi, Homayon Ghiasi

**Affiliations:** Center for Neurobiology and Vaccine Development, Ophthalmology Research, Department of Surgery, Cedars-Sinai Burns & Allen Research Institute, CSMC–SSB3, Los Angeles, CA, United States of America; Wayne State University School of Medicine, UNITED STATES

## Abstract

Complex interactions between HSV-1 and infiltrating immune cells play important roles in establishing localized, acute virus replication as well as chronic latent infection. The extent and duration of initial virus replication are the key determinants of subsequent pathologic inflammatory responses and therefore, the accumulation of immune cell populations at this time point is a key target for prevention. Therefore, we evaluated the role of various immune cell infiltrates between 1 h and 28 days post-infection (PI) using mice infected with virulent HSV-1 strain McKrae without corneal scarification. The effect of corneal scarification on immune cell infiltrates was also determined. We first determined the activation status and origin of macrophage infiltrates as early as 1 h PI. We found a sharp increase in the total macrophage population after 12 h PI, that was primarily due to infiltration of CCR2^+^ migratory macrophages, mostly in M1 status (MHC II^+^). The number of CCR2^-^ resident macrophages, mostly unpolarized (M0), increased gradually over time and peaked at 48 h PI. Interestingly, some of the resident macrophages gained an M2-like phenotype (CD206^Low^), which peaked at 12 h PI, concurrent with M1 macrophage infiltration. From 1–7 days PI, infiltration of various immune cells correlated strongly with HSV-1 replication, with neutrophils showing the biggest increase, and NKT cells the biggest decrease, after infection. The presence of geographical ulcer did not correlate with increased infiltration, while mice with corneal scarring had significantly more immune cell infiltration than those without corneal scarring. Overall, we showed time-dependent infiltration of various immune cells in the eye of HSV-1 infected mice. Initial infiltration of macrophages followed by infiltration of T cells at later times PI demonstrates the importance of targeting macrophages rather than other immune cells type, for therapeutic treatment of HSV-1.

## Introduction

It is well known that herpes stromal keratitis (HSK) mediated by herpes simplex virus type 1 (HSV-1) is an immunopathological disease and that immune cells play important roles in clearing the virus from the eye around days 6–7 post-infection (PI) [1]. HSK is the most common cause of vision impairment in humans, and occurs as a consequence of virus reactivation [2]. The extent and duration of immune cell infiltrates in the eye during both primary HSV-1 infection and reactivation can impact the severity of eye disease and the subsequent HSK, also is known as corneal scarring (CS) [3–11]. After ocular HSV-1 infection, innate immune cells are thought to play an important role in clearing virus from the eye. Recent studies showed that neutrophils, which start their response around 18 h PI, peak at day 2 PI, and eventually decline [12], along with other innate immune cells including NK cells, γ-delta T cells, macrophages, and dendritic cells (DCs), participate in virus clearance [13, 14].

Macrophages are known to be early-responders to virus infection [15–18]. Recently, macrophages and DCs were shown to be the main source of IL-1β and iNOS which, together with type 1 interferons, are essential to mount an immune response against HSV-1 infection [19]. From their resting state (M0), macrophages functionally polarize into either the pro-inflammatory (M1) or anti-inflammatory (M2) phenotypes depending on environmental cues [20–23]. Macrophages have been reported to become M1 polarized upon virus infection to help clear virus-infected cells from affected tissues by releasing pro-inflammatory cytokines, and then become M2 polarized to repair damaged tissues by releasing anti-inflammatory cytokines [22, 24–28]. We previously reported that HSV-1 infected mice, with macrophages altered toward the M2 phenotype by colony stimulating factor-1 (CSF-1) injection, showed less primary and latent infection than mice with macrophages altered toward the M1 phenotype by IFN-γ injection [26]. In addition, recombinant HSV-1 with constitutive expression of IL-4 (HSV-IL-4), which can alter macrophages toward M2 similar to CSF-1, also showed less local virus replication in the eye and less latency than parental virus or a recombinant HSV-1 expressing of IFN-γ (HSV-IFN-γ) [27].

These findings led us to investigate the role of M2 macrophages during early and late stages of ocular infection, in contrast to the general belief that M1 macrophages clear virus through a pro-inflammatory rather than an anti-inflammatory pathway. In addition to monitoring macrophage responses to infection, we also looked at various immune cell infiltrates in the cornea of infected mice. It is important to understand which type of immune cells are involved in initial virus clearing as a focus for developing immunotherapeutic methods. Our current study determined the origin and functional status of macrophage subtypes during the initial phase of virus infection. Following ocular HSV-1 infection, we tracked changes in other immune cell subtypes (T cells, DCs, B cells, monocytes, neutrophils, NK cells, NKT cells) up to 28 days PI. We compared immune infiltrates in infected mice with geographical ulcer (GU) or CS to those in healthy mice with infected corneas. Upon HSV-1 infection, but not in mock-infected animals, the total macrophage population increased (2-fold) in the cornea as early as 1 h PI. After infection, CCR2^+^ migratory macrophages were polarized to the M1 phenotype and CCR2^-^ resident macrophages remained mostly M0 (unpolarized), but almost 50% switched to the M2-like phenotype. By analyzing different immune cell populations, we found a general trend of expansion of neutrophils, CD4^+^ T cells, and monocytes whereas, NK cells, CD8^+^ T cells, and DCs remained constant with low levels of viral expression. In contrast to these cell populations, we observed fewer NKT cells in infected than in uninfected mice. Finally, we studied the effect of immune cell infiltration of the cornea in the presence and absence of cornel scarification and observed that corneal scarification even without infection increased cell infiltration to levels seen in McKrae-infected mice.

## Results

### Effect of ocular HSV-1 infection on macrophage subtypes in cornea of infected mice

We have previously reported the importance of macrophage polarization in controlling acute virus infection [16, 26, 27]. Altering macrophage polarization toward the M2 phenotype reduced both primary and latent infection while polarization toward the M1 phenotype aggravated the severity of infection [26]. To understand the dynamics of macrophage response at early stages of HSV-1 infection (from 1 h to 72 h PI), we compared the origin of macrophage infiltrates (tissue-resident vs migratory) as well as their activation status at the same timepoints. C57BL/6 mice were infected with 2 X 10^5^ PFU/eye of wild type HSV-1 strain McKrae without corneal scarification and mock-infected mice were used as controls. Corneas from both groups of mice were isolated at 1, 2, 3, 6, 9, 12, 24, 48, and 72 h post ocular infection and the corneas from each mouse were combined for FACS analysis as described in the Materials and Methods.

**Fig 1:**
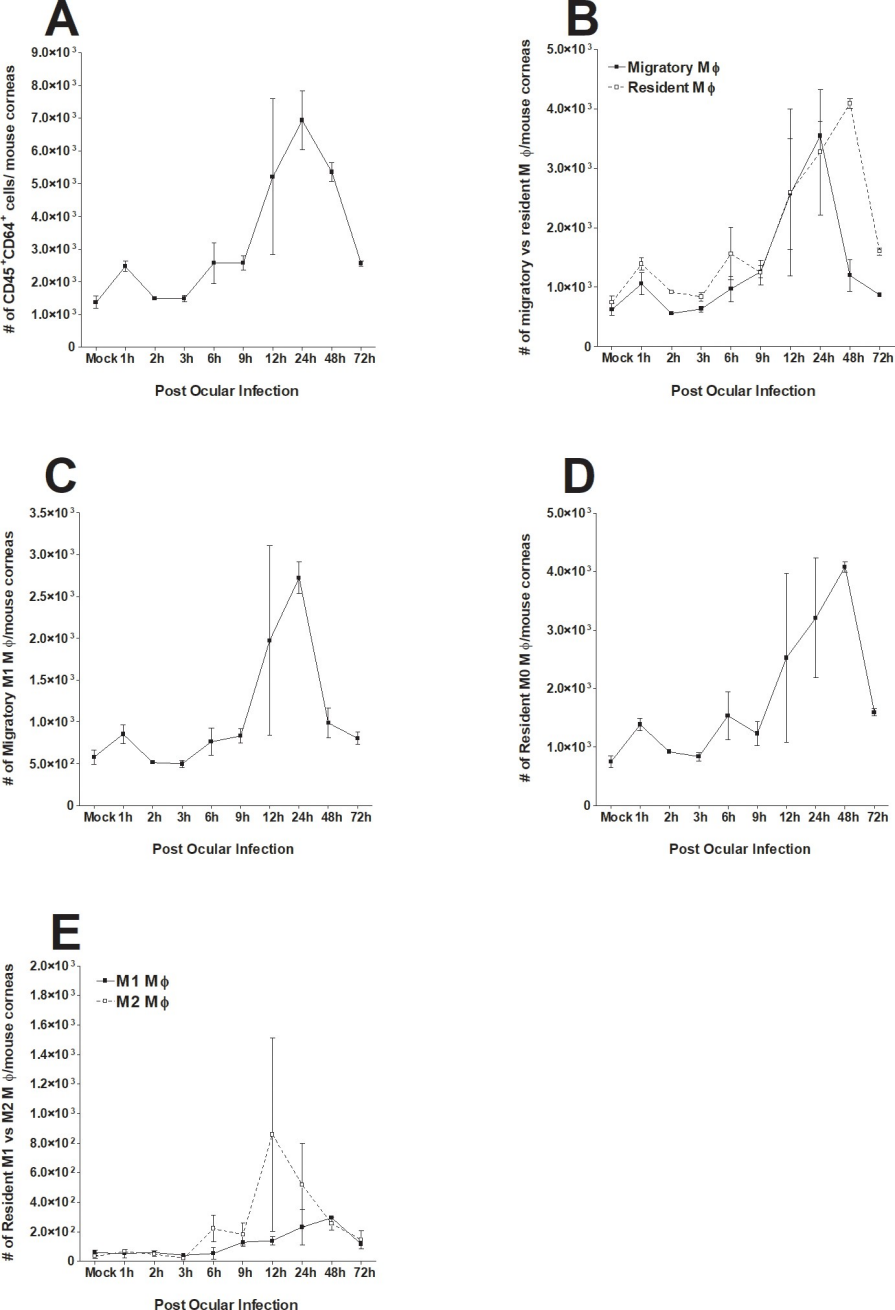
Monitoring of changes in macrophage subtypes in cornea of ocularly infected mice. Female C57BL/6 mice were ocularly infected with 2 X 10^5^ PFU/eye of HSV-1 strain McKrae. Corneas of infected mice were harvested at 1 h, 2 h, 3 h, 6 h, 9 h, 12 h, 24 h, 48 h and 72 h PI. Single cell suspensions of corneal cells were prepared and subjected to flow cytometry as described in Materials and Methods. Panels show the changes in the number of different macrophage subtypes at different times PI. Uninfected mice were used as mock control. For each time point we used a separate mock correspondent to the time that we performed the FACS analyses and due to their similarity in all tested time points, we only showed one mock in each figure. Each point represents mean cell number ± SEM per 3 mice corneas. Experiments were repeated three times. Panels: A) Total macrophages (CD45^+^CD64^+^); B) CCR2^+^ migratory and CCR2^-^ resident macrophages; C) Migratory M1 macrophages (CCR2^+^MHC-II^+^); D) Resident M0 macrophages (CCR2^-^MHC-II^-^CD206^-^); and E) Resident M1-like and M2-like macrophages.

The CD45^+^CD64^+^ total macrophage population had increased by 2-fold as early as 1 h PI while the control group did not (Fig 1A, p = 0.004). The total macrophage population continued to increase to 24 h PI, followed by a sharp decline by 48 to 72 h PI (Fig 1A). The number of CCR2^+^ migratory macrophages and CCR2^-^ resident macrophages increased similarly up to 24 h PI (Fig 1B). However, the number of CCR2^+^ migratory macrophages sharply declined between 24 and 48 h PI and continued to decline until 72 h PI. In contrast, CCR2^-^ resident macrophages increased up to 48 h PI, followed by a sharp decline by 72 h PI (Fig 1B). The polarization status of macrophage subtypes was also examined. At all-time points, CCR2^+^ migratory macrophages remained almost exclusively M1 polarized (MHC II^+^) (Fig 1C). In contrast, almost all CCR2^-^ resident macrophages remained unpolarized (M0) (MHC II^-^CD206^-^) at all time points (Fig 1D). Interestingly, a group of CCR2^-^ resident macrophages had an M2-like phenotype (CD206^Low^) upon HSV-1 infection (Fig 1E). The number of M2-like, CCR2^-^ resident macrophages reached its maximum (nearly 50%) at 12 h PI, and then gradually decreased over the remaining time course (Fig 1E).

In conclusion, we observed different dynamics of CCR2^+^ migratory and CCR2^-^ resident macrophages, and their polarization status upon HSV-1 infection. CCR2^+^ migratory macrophages remained almost exclusively M1, while CCR2^-^ resident macrophages remained almost exclusively M0 most of the time and half of the population had an M2-like phenotype at 12 h PI. Similar to migratory M1 macrophages, the number of M2 resident macrophages was maximal at 12 h PI, and quickly diminished thereafter.

### Detection of HSV-1 gC in the cornea of infected mice

**Fig 2:**
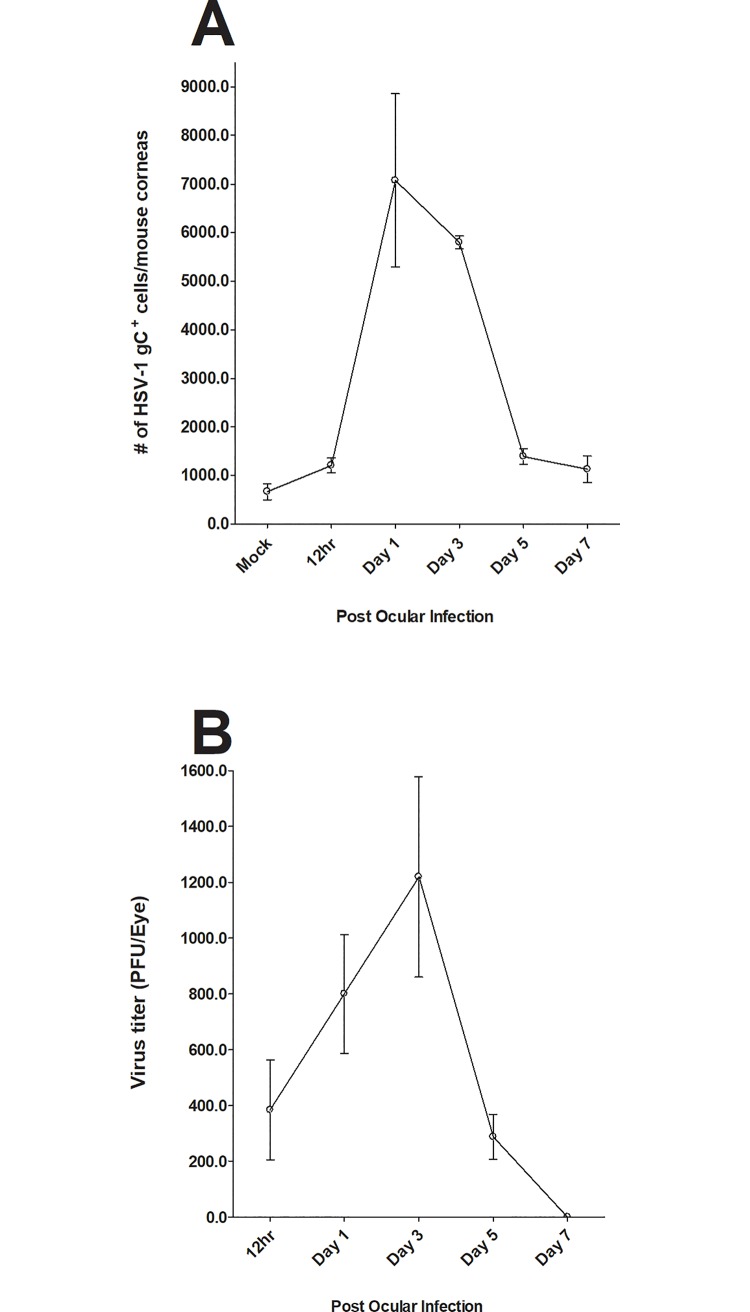
Monitoring the presence of virus in cornea of infected mice. Mice were ocularly infected with HSV-1 as described above and viral titers were measured in the eye of infected mice by plaque assay 12h, 1, 3, 5 and 7 days PI using eye swabs from 20 eyes. At the indicated time points, corneas of infected mice were isolated and single cell suspensions were prepared as described in Fig 1 above. Single cell suspension was stained with FITC anti-gC antibody. Each point represents mean cell number ± SEM per 3 mice corneas. Experiments were repeated three times. Panels: A) gC expression in cornea of infected mice: and B) Virus titer in the eye of infected mice.

To confirm the presence of viral protein in the cornea and HSV-1 in tears of infected mice, the presence of virus in the eye of infected mice was determined using a standard plaque assay at 12 h, 1, 3, 5, and 7 days PI. The presence of HSV-1 gC in corneas of infected mice was also evaluated at the same times by staining isolated single cell suspensions with anti-gC mAb. Mock-infected mice were used as controls. FACS analysis revealed the presence of gC expression in the cornea of infected mice (Fig 2A). gC expression increased from 12 h PI to day 1 PI and declined on days 3, 5, and 7 PI (Fig 2A). As expected, no virus was detected in mock-infected control group (Fig 2A). Similar analysis of eye swabs also showed the presence of infectious virus at 12 h, 1, 3, and 5 days PI but not day 7 PI (Fig 2B). While expression of gC in cornea of infected mice (Fig 2A) correlated with the presence of virus in tears (Fig 2B), peak level of gC expression did not correlate with the peak virus titers (Fig 2A vs. Fig 2B). These results confirmed the presence of infectious virus in the cornea of infected mice suggesting that the observed changes in immune cell infiltrates are due to the presence of virus and virus replication.

### Monitoring changes in various immune cell infiltrates in the cornea of infected mice at various times PI

**Fig 3:**
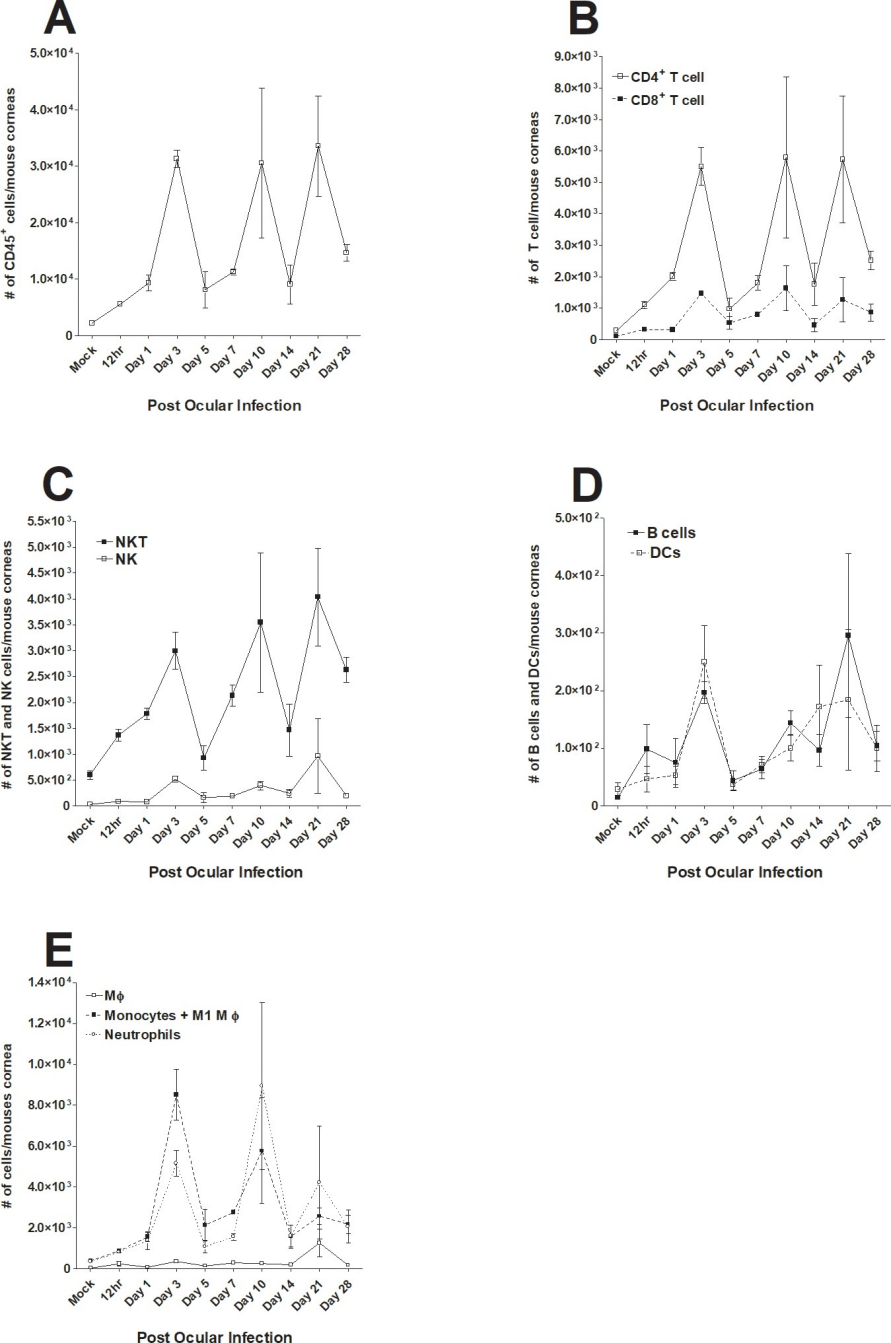
Effect of ocular HSV-1 infection on alteration of various immune infiltrates in cornea of infected mice at different times PI. Mice were ocularly infected with 2 X 10^5^ PFU/eye of HSV-1 strain McKrae. Corneas of infected mice were harvested at days 12 h, 1, 3, 5, 7, 10, 14, 21 and 28 days PI. Single cell suspension of corneal cells was prepared and subjected to flow cytometry as described in Materials and Methods. Panels show the changes in the number of different macrophage subtypes at different times PI. Uninfected mice were used as mock control. For each time point we used a separate mock correspondent to the time that we performed the FACS analyses and due to their similarity in all tested time points, we only showed one mock in each figure. Each point represents mean cell number ± SEM per 3 mice corneas. Experiments were repeated three times. Panels: A) CD45^+^ immune cells; B) CD4^+^ and CD8^+^ T cells; C) CD4^+^ to CD8^+^ T cell ratio; D) NK and NKT cells; E) B cells and DCs; and F) F4/80^+^Ly6C^-^ macrophages, F4/80^+^Ly6C^+^ monocytes and M1 macrophages, and F4/80^-^Ly6C^+^ neutrophils.

**Fig 4:**
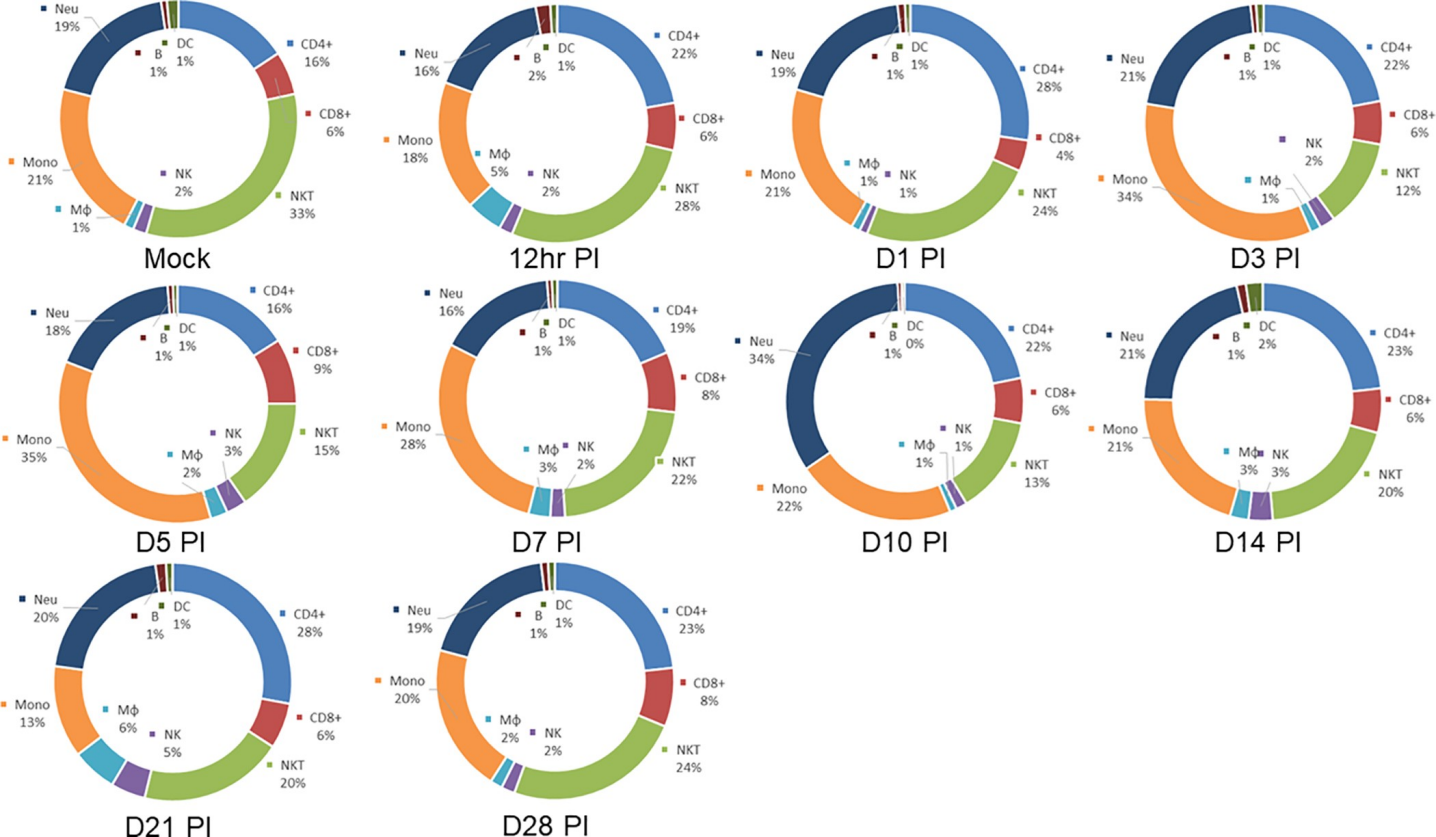
Pie charts showing the changes in relative percentages of immune cell subtypes in the cornea of infected mice. Data described in Fig 3 above were used to measure the relative percentage of CD4^+^, CD8^+^, NKT, NK, Mφ (macrophages), Mono (monocytes) including M1 macrophages, Neu (neutrophils), B (B cells) and DC.

In addition to determining the levels and status of macrophage infiltrates in the cornea of infected mice as we described above (Fig 1), we also monitored short-term and long-term dynamics of CD45^+^, CD4^+^, CD8^+^, NK, NKT, B cells, DCs, macrophages, monocytes, and neutrophil infiltrates following corneal infection and compared them with those of corneas from mock-infected mice. Mice were infected with McKrae and the corneas of infected mice were isolated on 12 h, 1, 3, 5, 7, 10, 14, 21, and 28 days PI. Single cell suspensions of corneas from infected and mock control mice were stained with each specific antibody as described in Materials and Methods and changes in each cell population were determined by flow cytometry (Fig 3). The number of CD45^+^ cells increased from 12 h to 3 days PI, followed by a cyclical decline on days 5 and 7 PI followed by expansion on day 10, decline on day 14, another expansion on day 21 and another decline on day 28 PI (Fig 3A). A nearly identical trend was observed for CD4^+^ and CD8^+^ T cells, with significantly higher numbers of CD4^+^ T cells than CD8^+^ T cells on all days except day 5 PI (Fig 3B). NKT cells also showed a nearly identical cyclical fluctuation as CD45^+^ and T cells and their numbers increased over time (Fig 3C). In contrast, the number of NK cells was lower than that of NKT cells even in the mock group and remained relatively constant throughout the experiment, peaking on day 21 PI (Fig 3C). The numbers of B cells and DCs were similar and remained stable from 12 h PI to 28 days PI (Fig 3D). The number of macrophages (F4/80^+^Ly6C^-^) was stable throughout the experiment (Fig 3E, MΦ). The patterns of F4/80^+^Ly6C^+^ monocytes, including M1 macrophages, and F4/80^-^Ly6C^+^ neutrophils were similar and the number of monocytes was significantly higher than that of neutrophils (Fig 3E). The relative ratios of each infiltrate to the mock group at various time points PI are shown in Fig 4. In summary, we observed cyclic fluctuations of some immune cell subtypes in corneas of infected mice at early times PI (up to day 7 PI) that roughly followed the pattern of acute viral replication in the eye.

### Effect of corneal disease on immune response in infected mice

**Fig 5:**
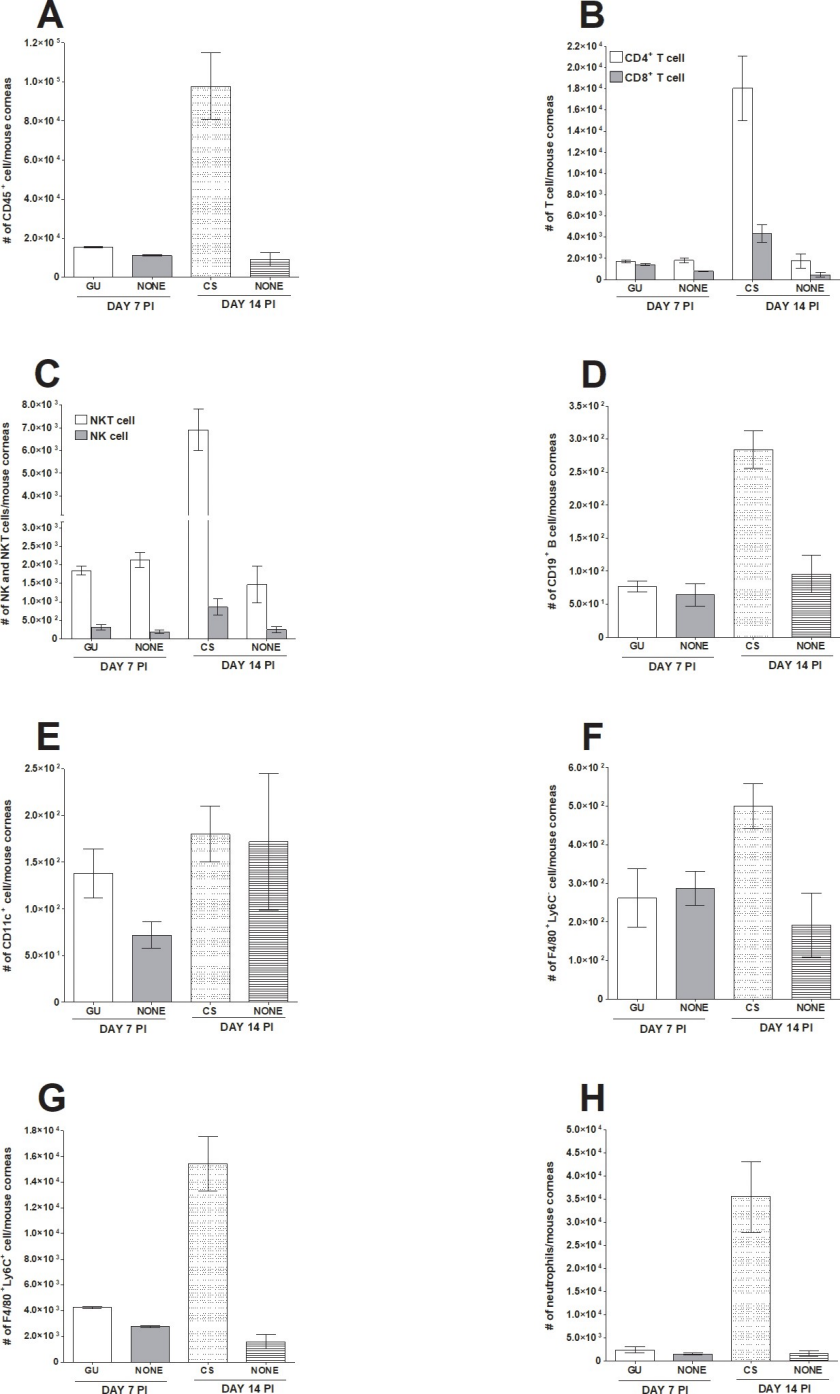
Comparison of infiltrating immune cells in the eye of infected mice with and without eye disease. Mice were ocularly infected as described in Fig 1 above. On day 7 and 14 PI, mice showing geographical ulcer (GU) or corneal scarring (CS), respectively were euthanized, corneas were collected, and subjected to flow cytometry and compared with mice that were similarly infected but did not show any eye disease (none). Each bar represents mean cell number ± SEM from 3 mice corneas. Experiments were repeated three times. Panels: A) CD45^+^ immune cells; B) CD4^+^ and CD8^+^ T cells; C) NK and NKT cells; D) CD19^+^ B cells; E) CD11c^+^ DCs; F) F4/80^+^Ly6C^-^ macrophages; G) F4/80^+^Ly6C^+^ monocytes and M1 macrophages; and H) Neutrophils.

**Fig 6:**
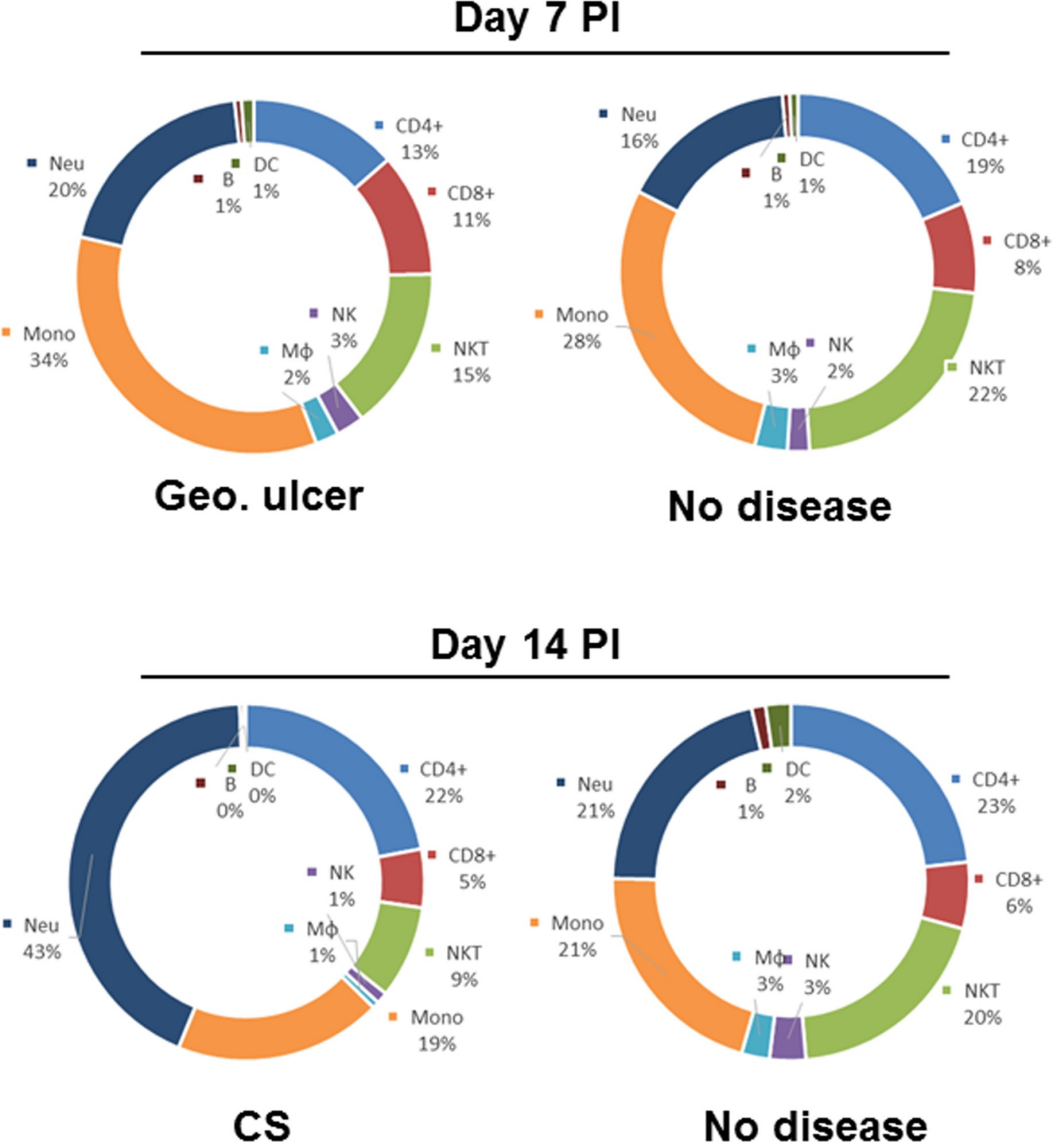
Pie charts comparing relative percentages of major immune cell subtypes in the corneas of infected mice between mice having geographical ulcer (GU), corneal scarring (CS) or no symptoms (NONE). Data described in Fig 5 above were used to measure the relative percentage of CD4^+^, CD8^+^, NKT, NK, Mφ (macrophages), Mono (monocytes) including M1 macrophages, Neu (neutrophils), B (B cells) and DC.

While evaluating corneal infiltrates at various times PI, we noted that on day 7 PI some infected mice showed signs of GU, while on day 14 PI some infected mice showed signs of CS. Because mice described above (Figs 3 and 4) had no signs of eye disease, mice with GU or CS were analyzed separately (Figs 5 and 6) and compared with their respective counterparts on day 7 or 14 that we described above. On day 7 PI, mice with GU and those with no eye disease (None) had similar levels of immune infiltration: CD45^+^ (Fig 5A, day 7 PI), CD4^+^ and CD8^+^ T cells (Fig 5B, day 7 PI), NKT and NK cells (Fig 5C, day 7 PI), CD19^+^ B cell (Fig 5D, day 7 PI), CD11c^+^ (Fig 5E, day 7 PI), F4/80^+^Ly6C^-^ (Fig 5F, day 7 PI), F4/80^+^Ly6C^+^ (Fig 5G, day 7 PI), and neutrophils (Fig 5H, day 7 PI). In contrast to mice with GU, on day 14 PI mice with CS had significantly more immune cell infiltration than did mice with no CS (Fig 5, day 14 PI). The number of CD45^+^ cells in mice with CS was 11-fold higher than in mice with no CS (Fig 5A, p = 0.03, day 14 PI). The number of CD4^+^ and CD8^+^ T cells was10-fold higher in mice with CS than in mice with no CS (Fig 5B, p<0.03, day 14 PI). Mice with CS had 5-fold more NKT cells than did mice with no CS (Fig 5C open bars, p = 0.01, day 14 PI), 3-fold more B cells (Fig 5D, p = 0.009, day 14 PI), 3-fold more F4/80^+^Ly6C^-^ macrophages (Fig 5F, p = 0.04, day 14 PI), 10-fold more F4/80^+^Ly6C^+^ monocytes including M1 macrophages (Fig 5G, p = 0.02, day 14 PI), and 22-fold more F4/80^-^Ly6C^+^ neutrophils (Fig 5H, p = 0.001, day 14 PI). In contrast, we found no significant difference between NK cells (Fig 5C filled bars, p = 0.1, day 14 PI) or DCs (Fig 5E, p = 0.9, day 14 PI) in mice with or without CS.

We also compared the overall immune cell profiles in mice with GU, CS, or with no eye disease (Fig 6). On day 7 PI, both neutrophils and monocytes including M1 macrophages were moderately higher in the GU group than in mice with no disease group (34% vs 28% and 20% vs 16%, respectively; Fig 6, day 7 PI). On day 7 PI, there were fewer NKT cells and CD4^+^ T cells in the GU group than in the no disease group. On day 14 PI, changes in the number of neutrophils was the most notable difference between the groups (Fig 6, day 14 PI). Mice with CS had twice as many neutrophils than did mice with no disease (Fig 6, day 14 PI, 43% vs 21%). In contrast, mice with CS had significantly fewer NKT cells than did mice with no disease (Fig 6, day 14 PI, 9% vs 20%). No notable differences were identified in other subtypes. In summary, we found no correlation between the presence of cellular infiltrates in mice cornea and the development of GU, but a significant correlation between the presence of infiltrates and the development of CS. Finally, our results suggest that HSV-1 infection have a suppressing effect on NKT cells.

### Effect of corneal scarification without infection on immune infiltrates

**Fig 7:**
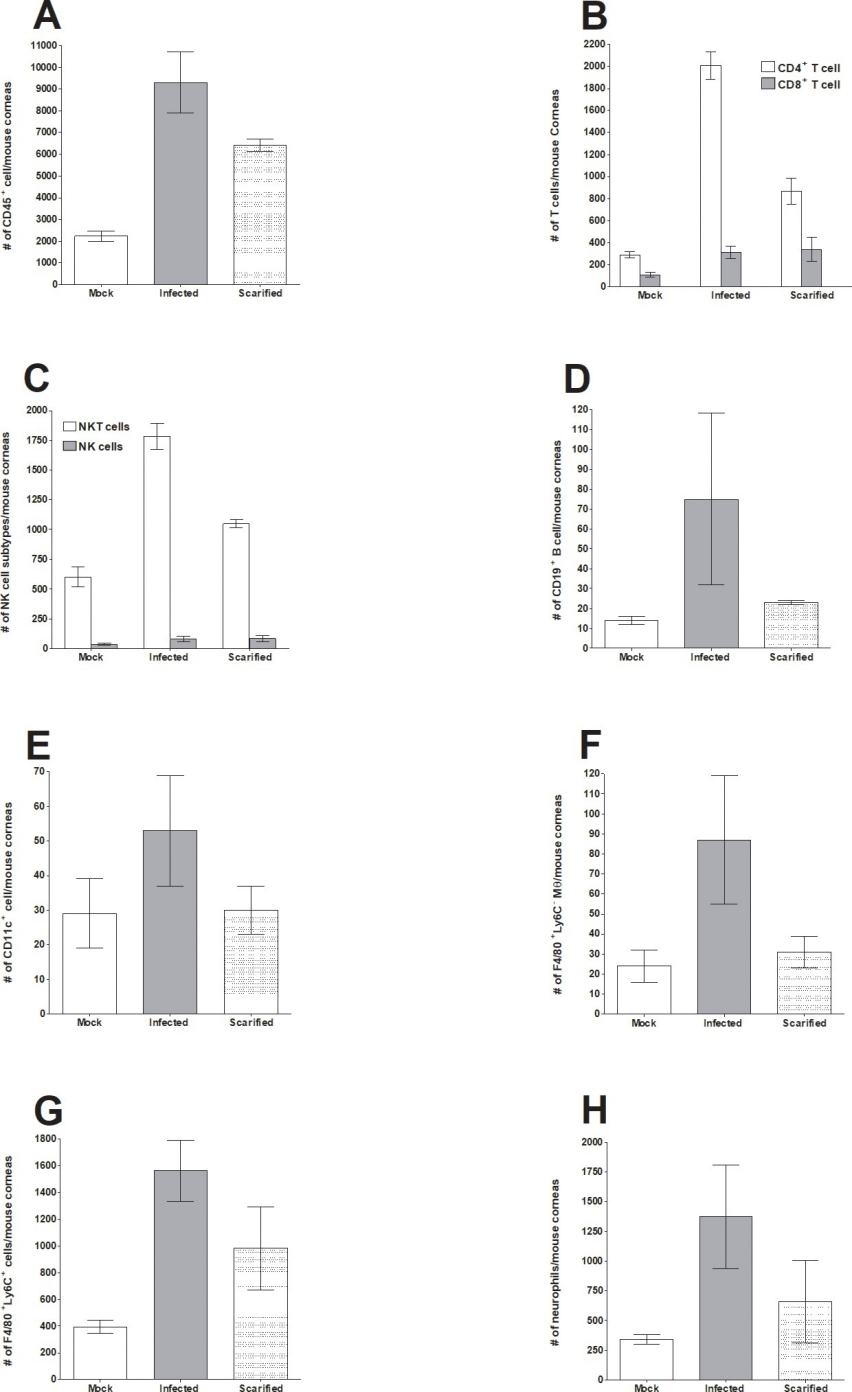
Effect of corneal scarification on infiltrating immune cells in the eye of mice on 24 h post treatment. Female C57BL/6 mice were ocularly infected as described in Fig 1 above without corneal scarification. A second group of mice were scarified and infected with HSV-1 as described in Materials and Methods and uninfected-unscarified mice were used as mock control. Twenty-four h later, mice were euthanized, corneas were collected, and subjected to flow cytometry as described in Fig 3 above. Each bar represents mean cell number ± SEM from 3 mice corneas. Experiments were repeated three times. Panels: A) CD45^+^ immune cells; B) CD4^+^ and CD8^+^ T cells; C) NK and NKT cells; D) CD19^+^ B cells; E) CD11c^+^ DCs; F) F4/80^+^Ly6C^-^ macrophages; G) F4/80^+^Ly6C^+^ monocytes and M1 macrophages; and H) Neutrophils.

McKrae is a virulent ocular isolate that does not require corneal scarification for efficient ocular virus replication and establishment of latency. In contrast, in many avirulent strains of HSV-1 such as KOS and RE, corneal scarification increases virus replication in the eye and latency in trigeminal ganglia. We did not use corneal scarification in the studies described above. Since mechanical damage of a tissue can induce an inflammatory response and subsequent wound healing process, we compared the effect of corneal scarification on immune cell infiltration in mice infected with McKrae without corneal scarification and an untreated control group. At 24 h post-corneal scarification or infection, mice were euthanized, corneas were collected, and subjected to flow cytometry analysis as described in Materials and Methods. Immune cell infiltration was compared in McKrae-infected mice and untreated mice (mock) at 24 h PI. We found that corneal scarification alone induced significant changes in some immune cell populations (Fig 7). Compared to mock, corneal scarification significantly increased the number of CD45^+^ cells to a level similar to McKrae-infected mice (Fig 7A, p = 0.02). Following scarification, CD4^+^ T cells increased by 3-fold over mock (Fig 7B, p = 0.006) but were lower than seen in McKrae-infected mice (Fig 7B). After corneal scarification, the increase in CD8^+^ T cells over the mock group was similar in infected and scarified cornea (Fig 7B). The number of CD19^+^ B cells (Fig 7D), DCs (Fig 7E), F4/80^+^Ly6C^-^ cells (Fig 7F), and neutrophils (Fig 7H) in scarified corneas were similar to those in mock controls. In contrast, scarified corneas had higher numbers of F4/80^+^Ly6C^+^ infiltrates than seen in mock controls, but similar numbers as seen in the infected group (Fig 7G). These results suggest that corneal scarification can alter immune infiltrates in the eye similar to that seen in infected mice and these changes may affect interpretation of the results.

## Discussion

HSK is the predominant cause of human blindness in the western world. To understand the pathology of the disease and to develop prevention strategies, it is essential to investigate the effect of HSK on the host immune response. It has been reported that higher numbers of immune cells in the eye correlate with increased pathogenesis [15, 17]. Virus strain, infection dose, mouse strain, and physical alteration of corneas affect HSV-1 pathogenesis. Initial viral replication in epithelial cells of the cornea begins with virus entry, which has detrimental effects on the host immune system. Innate immune cells play an important role in viral clearance, which is sustained by the adaptive immune response [12]. In humans, HSV-1 infections are difficult to study because primary infections are usually asymptomatic. Therefore, mouse models are an excellent way to study HSV-1 induced pathogenesis [29]. Recent studies have shown that HSK pathogenesis depends largely on immune responses generated by the host, but also depends on important factors like virus strain, viral clearance, and mice strain [30].

The objective of our study was to analyze the phenotype of immune cell infiltrates after ocular infection with HSV-1. Macrophages are believed to be one of the dominant infiltrates in the cornea after ocular HSV-1 infection [31, 32]. Macrophages depletion resulted in increased viral load in the eye of infected mice [33, 34]. We have correlated viral presence in the cornea with the time at which immune infiltrates enter the cornea. We found that neutrophils and macrophages play vital roles in viral clearance with macrophages starting their response as early as 1 h PI. This is similar to previous findings in which enhanced clearance of virus from the eye was mediated by macrophages [35]. To further clarify the role of macrophages in viral clearance and to determine whether macrophages are beneficial or detrimental to the host, we characterized the effect of macrophage polarization. The outcome of disease largely depends on the balance between migratory (M1) and resident (M2) macrophages in damaged tissue [36]. To profile macrophage dynamics upon HSV-1 infection, we compared our study with results of the Liu group, who studied macrophage characteristics in normal and injured corneas [37]. Although our model of ocular infection looks at different pathological conditions, we observed macrophage dynamics in HSV-1 infection that were very similar to those reported by Liu et al. upon corneal wounding [37]. In the Liu study, both CCR2^+^ and CCR2^-^ macrophages participated in corneal wound healing by balancing the inflammatory response. Liu et al hypothesized that CCR2^+^ migratory macrophages, mostly the M1 phenotype, promoted early inflammation after corneal wounding and that CCR2^-^ resident macrophages, mostly the M2 phenotype, inhibited inflammation to support wound healing. We also observed that almost all CCR2^-^ migratory macrophages were in M1 inflammatory status throughout the experiment and CCR2^-^ resident macrophages were mostly unpolarized (M0) or M2 polarized. Therefore, we agree with Liu’s hypothesis that initial infiltration of CCR2^+^ migratory macrophages may contribute to early inflammation.

In this study, we showed that the percentage of NKT cells declined following infection. NKT cells are known to protect against viral infection and cancer by modulating innate and adaptive immune responses via cytokine secretion [38], CD40 upregulation, and DC activation [39]. Related to this study, it has been shown that following murine cytomegalovirus (MCMV) infection, the liver NKT cell population is dramatically reduced [39]. NKT cells are known to undergo activation induced cell death (AICD), a process in which activation of cells is rapidly followed by apoptosis [40]. Similarly, the observed decline in NKT cells in infected cornea may be a result of NKT cells undergoing apoptosis. Studies in NKT-cell-deficient mice have shown that classical NKT cells do not play a critical role in early clearance of MCMV infection [41]. In contrast to the decline in NKT cells, our findings revealed that after HSV-1 infection, NK cells remained at a constant baseline level and thus, unlike CD4^+^ T cells, macrophages and neutrophils, NK cells may not have an overriding roles. In contrast to our study, NK cells have been shown to regulate inflammation and may prevent autoimmune diseases while a lack of NK cells enhanced the severity of experimental autoimmune encephalomyelitis (EAE) [42]. Furthermore, the NK cell response is robust in MCMV infected mice and helps to control infection by a perforin-dependent mechanism [43]. Published studies show that NK cells, but not NKT cells, are required to control the acute phase of MCMV infection in spleen and liver cells of infected mice [44]. We have also shown that NK cells play an important role in protecting naive C57BL/6 mice against CS and death following ocular HSV-1 infection [45].

DCs are well known to act as messengers between the innate and adaptive immune systems. It was previously believed that the cornea was an immune privileged site due to the absence of antigen-presenting cells (APCs) such as macrophages and DCs [46]. It was known that these cells enter the cornea from non-corneal tissues and that during inflammation, the number of DCs increase with MHC class II expression and upregulation of B7 costimulatory molecules CD80 and CD83 [46]. Later, Frank and colleagues reported an interplay between DCs, NK cells, and inflammatory monocytes during HSV infection [13]. They reported that early depletion of DCs up to 24 h PI delayed virus clearance from the cornea. DC depletion had no effect on extravasation or activation status of NK cells, inflammatory monocytes, or neutrophils, but did impede the migration of NK cells and inflammatory monocytes but not neutrophils toward the inflammatory site in the cornea. Chemokines involved in migration and activation of NK cells, macrophages, and monocytes showed reduced expression. A recent study of DCs that enter 24 h PI, unraveled their role in activating CD4^+^ T cells and their expansion [47]. Evaluating the role of resident corneal DCs that enter 7 days after corneal infection demonstrated that they are involved in clearing HSV by facilitating recruitment of NK cells and inflammatory monocytes to the infection site [13]. In contrast, our previous findings demonstrate that DC depletion results in an approximately 5-fold reduction of latent virus and significant reduction in viral reactivation, suggesting that CD8α^+^ DCs contribute to increased latency but not primary infection [48, 49]. Our study showed a constant baseline level of DCs after HSV-1 infection.

We also looked at T cell responses generated after HSV-1 infection and our experiments imply that CD4^+^ T cells play a crucial role in the HSV-1 pathogenesis. As stated in past studies, CD4^+^ T cells, particularly T_H_1 cells are the principal orchestrators of the disease [50], we observed increased numbers of CD4^+^, but not CD8^+^, T cells during the disease course. Neutrophil recruitment trend increased to a maximum on day 10 PI. As previously illustrated, CXCR2 knockout mice were more susceptible to HSK than WT mice, which supports the relevance of neutrophils in the early stages of infection [51]. It is well known that neutrophils contribute in two ways to HSK pathology. The early neutrophil wave may contribute to virus clearance along with other innate immune cells like NK cells, macrophages, and gamma delta T cells. The neutrophil peak, along with CD4^+^ T cell infiltration (particularly Th1 cells) may contribute to corneal damage [12]. Similar to DCs, B cells showed a similar trend in expression after HSV-1 infection. Monocyte numbers correlated with the presence of virus in the eye. Ly6C^hi^ inflammatory monocytes and neutrophils have been shown to correlate with clinical signs of HSE in mice [52]. The ly6C^lo^ monocyte population replaced ly6C^hi^ inflammatory monocytes and hence contributing to the resolution phase.

Overall, our results show differential expression patterns of various immune cell populations. We found that macrophages infiltrate the cornea as early as 1 h PI. We also showed that most macrophage infiltrates remained in a CCR2^+^ migratory state (M1 state), which accounts for the early inflammatory response. Remaining macrophages acquire a CCR2^-^ resident state (M2 state), which contributes to wound healing. DCs, B cells, and NK cells maintain constant baseline populations, while the NKT cell population declined after infection compared to mock animals. The monocyte population increased on days 3–5 PI but declined later. Adaptive immune response followed an expected trend in which the CD4^+^T cell population increased early after infection, while the CD8^+^T cell population remained constant throughout disease progression and did not change in frequency with or without infection. Neutrophil numbers were maximal on day 10 PI and they showed similar fluctuation on days 14, 21, and 28 PI as other cell types. When comparing GU (Day 7 PI) and CS (Day 14 PI) with no disease, neutrophil numbers were maximal on day14 PI compared to the no disease group. The fluctuations of different immune cells over the 28 days post infection could be due to subclinical reactivation of HSV-1 even after no virus is detected in tears of ocularly infected mice. Previously several groups reported spontaneous molecular reactivation in infected mice [53–56].

In summary, we found that among all immune cells, macrophages accumulate in the eye of HSV-1 infected mice as early as 1 h PI and thus modulating macrophage activity could be an effective immunotherapeutic approach. We also found that corneal scarification significantly affected the number and type of infiltrating immune cells and therefore, data generated by corneal scarification prior to HSV-1 infection should be interpreted with caution. Overall, our data shows that macrophages are early-responders to HSV-1 infection in the eye of infected mice and would be a potential candidate for developing immunotherapeutic approaches to control HSV-1 infection.

## Materials and methods

### Ethics statement

All animal procedures were performed in strict accordance with the Association for Research in Vision and Ophthalmology Statement for the Use of Animals in Ophthalmic and Vision Research and the NIH *Guide for the Care and Use of Laboratory Animals* (ISBN 0-309-05377-3). Animal research protocols were approved by the Institutional Animal Care and Use Committee of Cedars-Sinai Medical Center (Protocols #5030 and #5374).

### Virus and mice

Plaque-purified, virulent, wild type herpes simplex virus 1 (HSV-1) strain McKrae was used in the study. Female inbred C57BL/6J (6–8 weeks old) mice were obtained from the Jackson Laboratory (Bar Harbor, ME).

### Ocular infection with HSV-1

Briefly, mice were infected ocularly with McKrae at 2 X 10^5^ PFU per eye as an eye drop in 2 μl of tissue culture media as we described previously [26, 27]. Except in one of the study, no corneal scarification was performed prior to infection. Corneal scarification was performed as described previously [57, 58].

### Cells

Rabbit skin (RS) cells were used for preparation of virus stocks, culturing mouse tear swabs, and determining growth kinetics. RS cells were grown in Eagle's minimal essential medium (MEM) supplemented with 5% fetal bovine serum (FBS).

### Cell infiltration response

Corneas of the infected mice were harvested at regular intervals up to 28 days post-infection (PI). For macrophage (Mφ) profiling, eyes were harvested at 0 h, 1 h, 2 h, 3 h, 6 h, 9 h, 12 h, 24 h, 48 h, and 72 h PI. For overall immune cell profiling, corneas were harvested at days 0, 0.5 (12 h), 1, 3, 5, 7, 10, 14, 21, and 28 PI. At least three mice were harvested at each time point for statistical analysis. Mice were monitored daily for any signs of corneal ulcer and blepharitis. For any mice showing the symptoms, the degree was measured on a scale of 0 (no scarring) to 4 (severe ulcer) as we described previously [1], and the day PI was recorded for comparison analysis.

### Viral titers of infected mice

Tear films were collected from 20 eyes of mice per group on 12h, 1, 3, 5 and 7 days PI using a Dacron-tipped swab [26, 27]. Each swab was placed in 1 ml of tissue culture medium and squeezed, and the amount of virus was determined by a standard plaque assay on RS cells.

### Flow cytometric analysis

Mice were euthanized with CO_2_ overdose followed by cervical dislocation. After euthanasia, eyeballs were collected, and the corneal tissues were cut into pieces and digested with 0.2% Liberase (Sigma-Aldrich; Cat. No. 5401119001) in Hank’s Balanced Salt Solution (HBSS) at 37°C for 1 h. The digested tissues were filtered through 70 μm filter to obtain single cell suspension. Cells were washed with PBS and then resuspended in PBS. Cells were first stained with Live/Dead Fixable Red Dye (Thermo Scientific; Cat. No. L34971) on ice, in the dark, for 15 min. After washing with cell staining buffer (Biolegend; Cat. No. 420201), cells were blocked with Fc blocking antibody (BD; Cat No 553141) on ice for 15 min, followed by incubation with antibody cocktails on ice, in the dark, for 30 min with Brilliant Stain Buffer (BD Biosciences; Cat. No. 563794). Antibodies used in the experiments are: For 6-color Mφ panel, (1) Pacific Blue anti-mouse CD45 (Clone 30-F11) (Biolegend, Cat. No. 103125); (2) BV421 anti-mouse CD64 (Clone X54-5/7.1) (Biolegend, Cat. No. 139309); (3) BV650 anti-mouse CCR2 (Clone SA203G11) (Biolegend, Cat. No. 150613); (4) PE anti-mouse CD206 (Clone C068C2) (Biolegend, Cat. No. 141705); (5) BV711 anti-mouse MHC II (Clone M5/114.15.2) (Biolegend, Cat. No. 107643). For 11-color immune cell panel, (1) Alexa Fluor 532 anti-mouse CD45 (Clone 30-F11 (Thermo Scientific; Cat. No. 58-0451-80); (2) BV650 anti-mouse Ly6C (Clone RB6-8C5) (Biolegend, Cat. No. 108441); (3) BV711 anti-mouse F4/80 (Clone BM8) (Biolegend, Cat. No. 123147); (4) PE-Cy5 anti-mouse CD3 (Clone 500A2) (BD Biosciences, Cat. No. 560773); (5) BV570 anti-mouse CD4 (Clone RM4-5) (Biolegend, Cat. No. 100541); (6) BV785 anti-mouse CD8 (Clone 53–6.7) (Cat. No. 100749); (7) PerCP-Cy5.5 anti-mouse NK1.1 (Clone PK136) (Biolegend, Cat. No. 108727); (8) Pacific Blue anti-mouse CD19 (Clone 6D5) (Biolegend, Cat. No. 115526); (9) BV421 anti-mouse CD11c (Clone N418) (Biolegend; Cat. No. 117329); and (10) FITC anti-gC (Genway, #20-902-170310). After incubation, cells were washed twice with cell staining buffer and subjected to flow cytometry analysis using SA3800 Sony Spectral Analyzer (Sony Biotechnology, San Jose, CA). Gating strategies are shown in S1 and S2 Figs.

### Statistical analysis

For all statistical tests, p-values less than or equal to 0.05 were considered statistically significant and marked by a single asterisk (*). P-value less than or equal to 0.001 were marked by double asterisks (**). Two-tailed student t-test with unequal variances was used to compare difference between two experimental groups. One-way ANOVA test was used to compare difference among three or more experimental groups. All experiments were repeated at least three times to ensure accuracy.

## Conclusion

Effective therapies for HSV-1 infection in the eye are lacking, despite knowledge that various immune cell infiltrates are involved in protection and disease following ocular HSV-1 infection. We identified various immune cell infiltrates in the eye of ocularly-infected mice and their presence was time dependent. Among these infiltrates, macrophages accumulated in the eye of HSV-1 infected mice as early as 1 h PI, thus modulating macrophage activity could be an effective immunotherapeutic approach to control HSV-1 associated eye disease. We also found that corneal scarification significantly affected the number and type of infiltrating immune cells. Therefore, data generated with corneal scarification prior to HSV-1 infection should be interpreted with caution.

## Supporting information

S1 Fig Gating Strategy of 6-color macrophage panel on day 21 post infection. (PDF)

S2 Fig Gating Strategy of 9-color immune cell panel on day 21 post infection. (PDF)
