# Supplementary figures and images for: CCR2+ migratory macrophages with M1 status are the early-responders in the cornea of HSV-1 infected mice
Source: PLoS One. 2019 Apr 18;14(4):e0215727. doi: 10.1371/journal.pone.0215727 (PMC6472814; doi:10.1371/journal.pone.0215727)

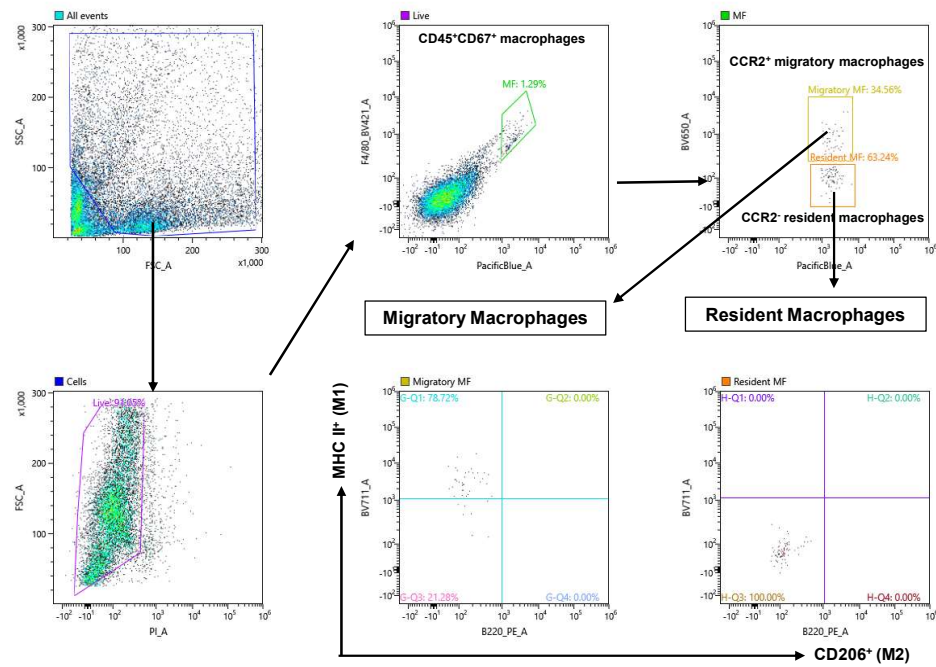

**S1 Fig.** Gating Strategy of 6-color macrophage panel on day 21 post infection.

Supplement: S1 Fig — (PDF) [file pone.0215727.s001.pdf]

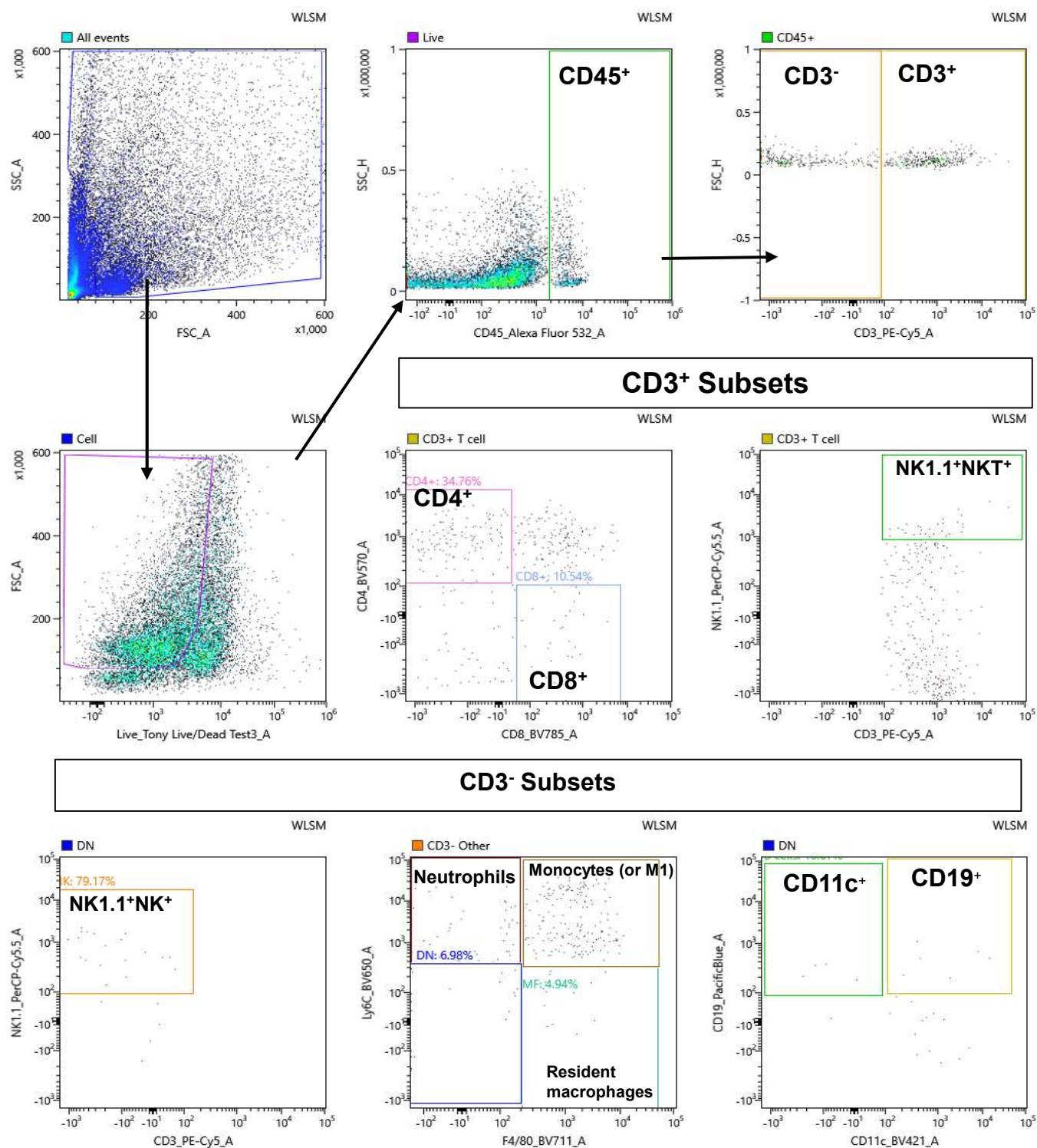

**S2 Fig.** Gating Strategy of 9-color immune cell panel on day 21 post infection.

Supplement: S2 Fig — (PDF) [file pone.0215727.s002.pdf]
